# An Experimental Investigation on the Creep Behavior of Deep Brittle Rock Materials

**DOI:** 10.3390/ma15051877

**Published:** 2022-03-02

**Authors:** Haozhe Chen, Zhushan Shao, Yoshiaki Fujii

**Affiliations:** 1 School of Civil Engineering, Xi’an University of Architecture & Technology, Xi’an 710055, China; chenhaozhe515@foxmail.com; 2 Shaanxi Key Lab of Geotechnical and Underground Space Engineering, Xi’an University of Architecture & Technology, Xi’an 710055, China; 3 School of Science, Xi’an University of Architecture & Technology, Xi’an 710055, China; 4 Laboratory of Rock Mechanics, Faculty of Engineering, Hokkaido University, Sapporo 060-8628, Japan; fujii6299@frontier.hokudai.ac.jp

**Keywords:** creep, brittle rock, moisture, digital image correlation (DIC), acoustic emission (AE)

## Abstract

The stability of deep rock engineering, especially during the excavation, is inextricably linked to the time-dependent mechanical properties of brittle rock. Therefore, the uniaxial creep test in a multilevel loading path is carried out, accompanying the real-time DIC (digital image correlation) and AE (acoustic emission) technologies. For the quartz sandstone, the lateral strain is more sensitive to increasing stress levels, and the lateral ductility is more significant during the creep process. The saturated quartz sandstone shows a certain bearing capacity before the volumetric dilation predominance. The softening effect of moisture causes a nearly invariable Poisson’s ratio during the middle stress stages, as well as the more notable increasing trend of a steady creep rate with an increasing stress level, reflected by the larger slope and the intercept in the fitting relations. The main shear pattern and the combination of the shear and splitting failures are separately shown by the dry and saturated quartz sandstone. For the granite, both compression and extension exist in the creep deformation, and the failure may first occur in the prominent deformation area with a cracking noise. The AE hits present a similar time-dependent behavior to the strain of rock, and the attenuation trend happens in both the AE amplitude and energy before the rock enters the unsteady phase. The incomplete specimen of granite exhibits a lower strength and a larger deformation, owing to the more remarkable damage accumulation reflected by the spatial distribution of the AE event points.

## 1. Introduction

As a typical and complicated dynamic disaster, the rockburst normally emerges in a sudden way during excavations in rock engineering, such as in tunnels at a great depth [1,2,3]. Numerous documents and field monitoring data have demonstrated that the most rockbust events occurred over a period of time after the excavation unloading, which is referred to as the time-delayed rockburst [4,5,6]. For instance, the delayed duration before the rockburst event in the Qinling Cuihua Mountain Extra-Long Tunnel reached 3–5 h [7] and, for the auxiliary tunnel and the diversion tunnel of Jinping II hydropower station, the occurrence of the rockburst experienced a delay ranging from several hours to days, and even months [6,7,8].

It is known that the time-delayed rockburst has a substantial connection with the evolution from time-dependent behavior to the creep failure of the surrounding rock [8,9]. Plenty of studies on the creep mechanical properties of the brittle rock material was through indoor experiments from various angles [10,11,12,13,14]. Kranz et al. (1982) found that the increasing temperature accelerates the creep failure and weakens the strength of granite using the static fatigue test [15]. Fujii et al. (1999) explored the effect of confining pressures and moisture on the circumferential strain behavior of granite and sandstone, using the short-term triaxial creep test, and believed that the circumferential strain can be applied in order to describe the damage during the test [16]. Okubo et al. (2008) designed the transparent triaxial cell to observe the time-dependent deformation of tuff and shale more intuitively [17]. Nicolas et al. (2017) analyzed the brittle creep mechanical behaviors of limestone by the evolution laws of ultrasonic wave velocities, as well as microstructural observations using the scanning electron microscope [18].

A dynamic non-destructive testing (NDT) method, called AE (acoustic emission) technology, achieved the widespread adoption for laboratory tests, which can monitor the failure process by recording the AE signals from the interior of the rock in real time [19,20,21,22,23,24]. Based on the creep loading results, Lin et al. (2009) analyzed the impact of confining pressures and temperature on the time-dependent strength degradation and damage evolution of granite by the variation of the AE cumulative counts and the AE rate [25]. Shi et al. (2018) obtained the damage accumulation process inside the cuboid-shaped sandstone specimen with pre-existing cracks of unequal lengths by comparing them with the spatial development of the AE event points [26]. Liu et al. (2019) described the time-dependent AE attenuation characteristics of granite, mainly via the change in the AE hits recorded in the uniaxial creep tests [27]. In addition, the application of the DIC (digital image correlation) technology, which is an optical non-contact three-dimensional deformation measurement approach, was used by scholars and engineers to study the time-dependent behavior of brittle rocks [28,29].

In this study, two deep rock samples of quartz sandstone and granite were separately gathered from the Laobishan Tunnel and the headrace tunnel in the Qinling Mountains and were used for the multilevel-unconfined creep tests. The effect of moisture on the time-dependent behavior and failure patterns of quartz sandstone was discussed, and by combing the 3D-DIC and the AE technologies, the creep characteristics of the surface deformation and the internal damage of granite were analyzed.

## 2. Experimental Procedure

### 2.1. Rock Specimen

**Figure 1:**
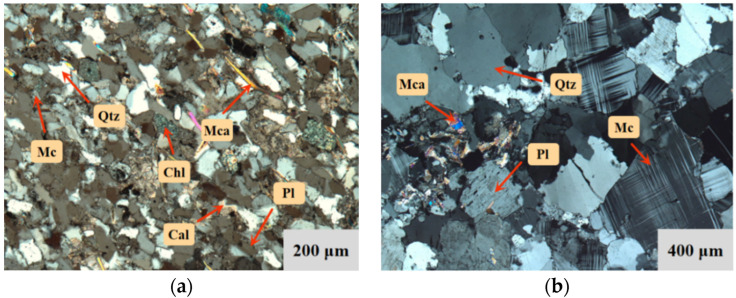
Thin sectional image of specimen meso-structure. (**a**) Quartz sandstone. (**b**) Granite.

Two types of brittle rocks that were taken from deep tunnels were adopted for the creep experiment. The fine-grained quartz sandstone mainly consisted of quartz (65%) and plagioclase (15%), with a small (4%) amount of muscovite, biotite, chlorite (3%), and metallic minerals (3%). The particle size ranged from 0.05 to 0.1 mm (Figure 1a). The medium-fine-grained granite was composed of 35% microcline, 30% quartz and plagioclase, and a little mica, of which the grain size primarily ranged from 0.2 to 1 mm (Figure 1b). The quartz sandstone was considerably hard and compact, which becomes more palpable for the granite.

Rock cores for quartz sandstone were promptly sealed and made into standard cylindrical specimens of 50 mm × 100 mm (diameter × height), and the granite specimen had a diameter of 40 mm and the same height. The specimens were divided into two groups by the rock type, and the group of quartz sandstone took into consideration the moisture condition. To minimize the influence of the original differences among the samples made by natural rock materials, the specimens extracted from the same rock core were set in the same test group.

The dry specimens were kept in an oven at 110 °C for 24 h. The saturated specimens were fully immersed for 30 days, and the water had been exposed to the air for 48 h to balance the pH value in the laboratory environment before the immersion. The group of granite was made into the dry specimen to reduce the moisture effect for as long as possible.

### 2.2. Test Setup

**Figure 2:**
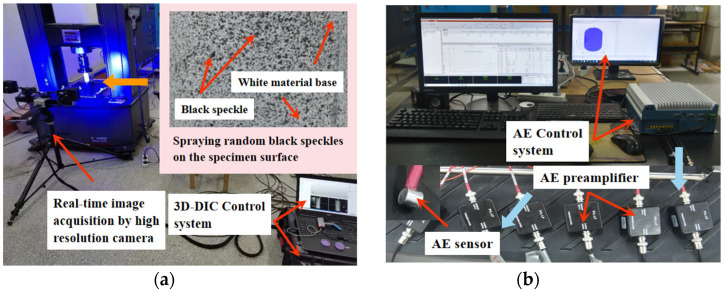
Diagram of DIC and AE technical system composition. (**a**) 3D-DIC system. (**b**) AE system.

The creep loading test in the unconfined condition was achieved by the servo-controlled rheology instrument manufactured by MTS Corporation, whose pressure was automatically regulated by the computer, and both load control and deformation measurement errors were smaller than 0.5%. Two real-time monitoring systems were separately performed on the creep experiments, where the 3D-DIC technology was used to attain the intuitive testing results concerning the creep deformation on the surface of the rock (Figure 2a). Before the operation, black speckles were randomly sprayed on the specimen, and there was no need for any further surface treatment because of the natural stochastic character of the rock material surface. The speckles should be adapted to high temperatures and a large range of deformations.

The three-dimensional AE system of the PCI-II model was applied to collect and process acoustic wave signals from the interior rock and the AE hits, AE energy, and AE amplitudes were calculated and recorded in real time (Figure 2b), reflecting the internal cracking development on a microscopic scale. For the sake of presenting the spatial distribution of the AE event points during the creep process from the deformation to failure, the six AE sensors, each with a 40 dB preamplifier with a frequency range between 100 kHz and 400 kHz, were attached to the specimen surface, and the sampling rate was 1 M s^−1^.

### 2.3. Testing Methods

**Figure 3:**
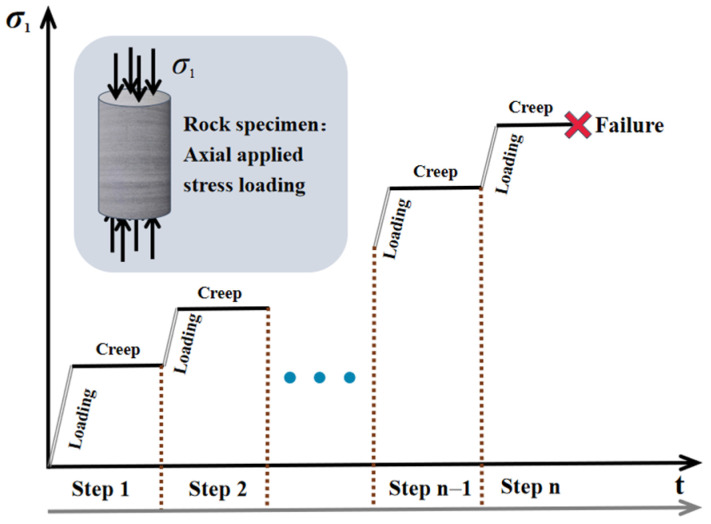
Schematic of multilevel-unconfined loading path.

The uniaxial creep tests were conducted in a multi-loading path (see Figure 3) and the loading rate for the stress increment was set at 0.01 mm/s in the displacement control mode. Before introducing the DIC system, the surface of the specimen should be clean and any bright light near the specimen is forbidden, as it will interfere with the interaction between the black speckles and the camera. As a base for the black speckles, the white material should be first sprayed on the specimen surface in a spacious and airy place. When the AE system was combined, the high vacuum grease was smeared between the specimen surface and the sensor to ensure a good coupling effect, and the sensor was fixed to the rock surface in line with the specific location in the 3D rectangular coordinates system, which was used to record the real-time spatial distribution of the AE event points.

## 3. Characteristics of Time-Dependency

### 3.1. Axial, Lateral, and Volumetric Creep Strain

**Figure 4:**
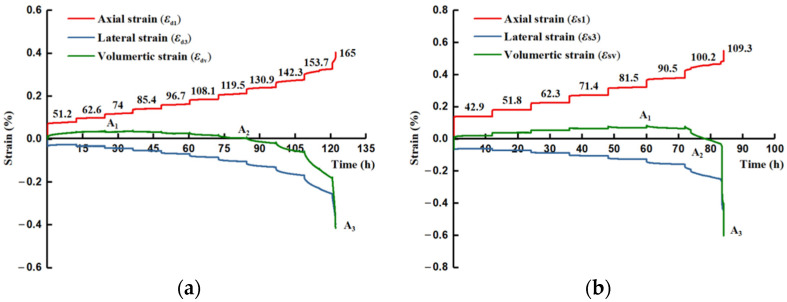
Axial, lateral, and volumetric creep strain curves of quartz sandstone under dry and saturated conditions. (**a**) Dry specimen. (**b**) Saturated specimen.

For the dry and saturated specimens (#Q-D and #Q-S) of quartz sandstone, the evolution in the axial and lateral strains with the multistage stresses is shown in Figure 4. Under low stress levels, the instantaneous elastic strain appeared after loading, and then the rock enters into the attenuated and stable stages when the strain rate decreases to an almost constant rate within a short time frame, manifesting as the primary and secondary creep. This similar variation mode continues until the axial and lateral deformation increases rapidly at a high stress level and the specimen ruptures in a violent way during the accelerating creep phase.

Figure 4 shows that the axial strain is more predominant than the lateral strain during the first few low load phases, which gradually reverses until the latter plays a leading role, especially at the last applied stress stages. The primary and secondary creep gradually becomes evident with the stress level, which is clearer for lateral strain. These phenomena indicate that the lateral deformation is more sensitive to the applied stress until the end of failure during the creep process, and the lateral ductility is more significant for the brittle rock under the time effect. The failure strength of #Q-D in a dry state observably exceeds the saturated specimen #Q-S, and the difference reaches about 55 MPa. Instead, the #Q-S, overall, displays a more palpable axial deformation than the #Q-D, as well as the approximate lateral development with #Q-D, and the increasing trend of the transient creep duration is more distinct for the #Q-S, suggesting that the softening effect caused by moisture accelerates the process from creep deformation to failure, exhibiting more ductility for the time-dependent behavior of brittle rock.

The volumetric strain *ε*_v_ is able to further depict the time-dependent deformation variation and is calculated from the axial strain *ε*_1_ and the lateral strain *ε*_3_ as:

(1) $$\varepsilon_{v}=\varepsilon_{1}+2\varepsilon_{3}$$

The creep volumetric evolvement contains two segments: compaction and expansion (see Figure 4). The compaction is predominant before reaching the positive maximum of the strain, marked A_1_, and due to the stronger leading tendency of dilation, the volumetric strain gradually declines to A_2_, demonstrating the relative balance between compaction and expansion. When the negative maximum A_3_ is achieved, the rock fails in a moment. It can be observed that the #Q-S experiences the longer duration of the compaction advantage until the creep load at 90.5 MPa, and A_1_ for the #Q-S is larger than the #Q-D, showing a certain payload capacity with a more compressible ductile deformation space under the impact of moisture. However, the volumetric dilation of the #Q-S displays more dominance than the #Q-D after A_1_, which leads to the greater and faster expansion of rupture at the last two stress stages. The phenomena can also illustrate why the strength of the dry rock specimen is mostly higher than that in the saturated state.

### 3.2. Evolution Laws of Poisson’s Ratio

**Figure 5:**
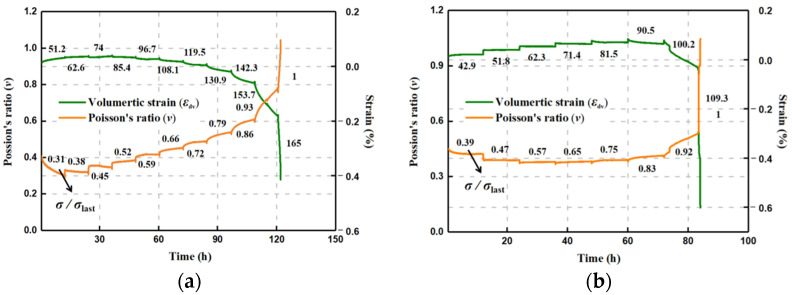
Time-dependent evolution of Poisson’s ratio and volumetric strain of quartz sandstone under dry and saturated conditions. (**a**) Dry specimen. (**b**) Saturated specimen.

Based on the above analysis, the relationship between the axial and lateral strains can also be described by Poisson’s ratio: ν = −*ε*_3_/*ε*_1_. Given the change of volumetric deformation, Figure 5 shows the variation in Poisson’s ratio during the creep loading. The evolution curves of the volumetric strain and Poisson’s ratio display an approximate symmetrical manner. The variation in Poisson’s ratio at the stage of loading is relatively small, and the loading of Poisson’s ratio changes with various stress levels. For the specimen #Q-S, the decreasing tendency of Poisson’s ratio commences with 0.39 *ε*_last_ until the notable increase emerges after the fixed load at 100.2 MPa. The applied loading duration lasts for 78.5 h when the creep of Poisson’s ratio becomes larger than 0.5, which basically corresponds to the mark A_2_ of volumetric strain, where the lateral deformation has started a governing pattern after the shift from volumetric compression to expansion (Figure 4). Under the dry condition, the #Q-D presents the continuous increase in Poisson’s ratio, except for the first stress phase (0.31 *ε*_last_), which primarily results from the ruling volumetric dilation with the applied load, especially when the applied stress reaches 130.9 MPa after about 85 h, which is also almost consistent with the time point A_2_ during the volumetric deformation evolution.

It is seen that the Poisson’s ratio of the #Q-S keeps a relatively invariable at each creep load from 0.47 *σ*_last_ to 0.75 *σ*_last_ and is nearly unaltered between the stress stages at 51.8 MPa and 81.5 MPa, which suggests, again, that the action of moisture leaves a certain space for the relative balance between axial and lateral deformation magnitudes. Before the volumetric expansion predominance, a certain bearing capacity for the saturated rock can remain, owing to the softening effect.

### 3.3. Evolution Laws of Creep Rate with Stress Levels

**Figure 6:**
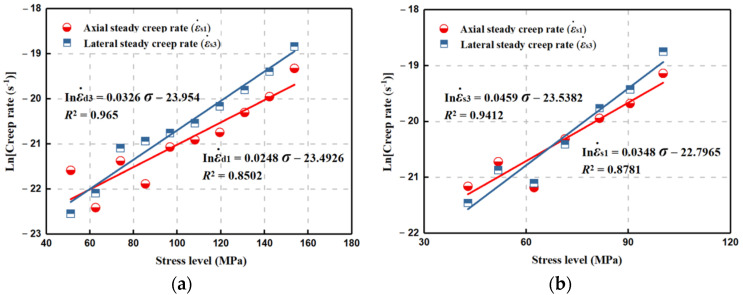
Relationship between steady creep rate and stress level for dry and saturated quartz sandstone specimens. (**a**) Dry specimen. (**b**) Saturated specimen.

The strain rate during the secondary creep is a crucial indicator to estimate the long-term stability and creep amount of the rock mass. As depicted in Figure 6, both axial and lateral steady creep rates basically increase with the multilevel stresses. The irregular evolvement is observed for both the #Q-D and #Q-S specimens during the first several low-applied stresses, which may result from the local heterogeneity differences in the rock, and then the lateral creep rate is continually higher than the axial one.

In order to minimize the impact on further analyses, the creep rate at the last stress level is not considered because of the strikingly rapid increase that marks the upcoming creep failure of rock. The stress corrosion, due to the subcritical crack growth, which might be the dominant mechanisms for the time-dependent deformation of such a brittle rock, is taken into account [30]. The relationship between the crack velocity *V* and the stress intensity factor *K*_I_ can be expressed by:

(2) $$V=V_{0}a\left(H_{2}O\right)exp\left(\frac{-E_{act}+\alpha K_{Ι}}{RT}\right)$$

(3) $$\alpha =\frac{2V^{*}}{\sqrt{\pi r_{c}}}$$

where *V*_0_ is a constant dependent of the material and environmental conditions, *a*(H_2_O) is the activity of water (approximately equal to the relative humidity), *E*_act_ is the activation energy per mol, *R* is the gas constant, *T* is the absolute temperature, V^*^ is the volume change from the original system to the activated complex per mol, and *r*_c_ is the radius of the curvature of the crack tip [31].

From Equation (2),

(4) $$lnV=lnV_{0}+lna\left(H_{2}O\right)+\frac{\alpha K_{Ι}}{RT}-\frac{E_{act}}{RT}$$

Assuming

(5) $$\dot{\varepsilon }=C_{1}V$$

and

(6) $$K_{Ι}=C_{2}\sigma$$

Hence, the steady creep rate $\dot{\varepsilon }$ is derived as follows:

(7) $$ln\dot{\varepsilon }=ln\dot{\varepsilon_{0}}+\ln a\left(H_{2}O\right)+\frac{C_{2}\alpha \sigma }{RT}-\frac{E_{act}}{RT}=\frac{C_{3}}{\sqrt{r_{c}}}\sigma +lna\left(H_{2}O\right)+C_{4}$$

As all the correlation coefficients (*R*^2^) surpass 0.8, the above model fits well the relationship between the steady creep rate and applied stress (see Figure 6). It is found that the forepart of the fitting curve for the axial relationship is larger than the lateral case, because of the variable relationship between the axial and lateral steady creep rates, reflecting the compression deformation advantage at low fixed loads to some extent. According to Table 1, *C*_3_/*r*_c_^1/2^ of the lateral fitting relationship is larger for both #Q-S and #Q-D, where *C*_3_/*r*_c_^1/2^ in the lateral case for the #Q-S is the maximum and is close to the #Q-D in the axial case. In*a*(H_2_O) + *C*_4_ in both the axial and lateral fitting expressions for the #Q-S is larger than that for the #Q-D, which is essentially consistent with the increasing trend described by Atkinson [32], where the *K*_I_-log*V* curve moves up with increasing *a*(H_2_O). Likewise, the results suggest that the influence of moisture is a substantial cause of the promotion of the creep failure of brittle rock, and matches the above comparison analysis on the time-dependent behavior differences between dry and saturated specimens.

### 3.4. Characterization of Time-Dependent Properties by DIC Technology

**Figure 7:**
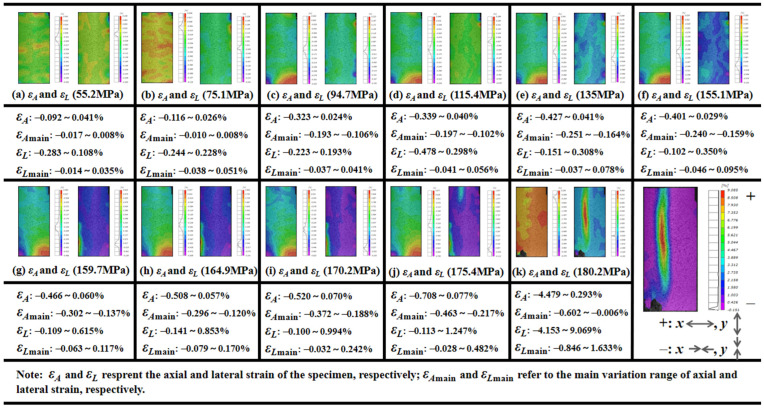
Variation range of axial and lateral creep strain with stress level for granite.

The visual evolution of the creep deformation on the specimen surface of granite (#G-D-1) is obtained by the 3D-DIC testing system, and the variation of the axial and lateral creep strain under multistage loading is exhibited in Figure 7. The strain in *x* and the *y* direction represents the axial and lateral deformation, respectively. Both the increasing tendency of the deformation and the relationship between axial and lateral strain are similar to the quartz sandstone specimen by observation. Although the local heterogeneity, such as the area near the top and bottom ends of the specimen, induces obvious deformation, on the whole, the lateral deformation is still greater than the axial development and this is clearer in the main variation range of strain. It can be seen that both positive and negative strains arise at each stress stage, indicating that both the deformation of the compression and extension exist on the rock during the creep process, and the fracture may likely happen in the distinct deformation position.

**Figure 8:**
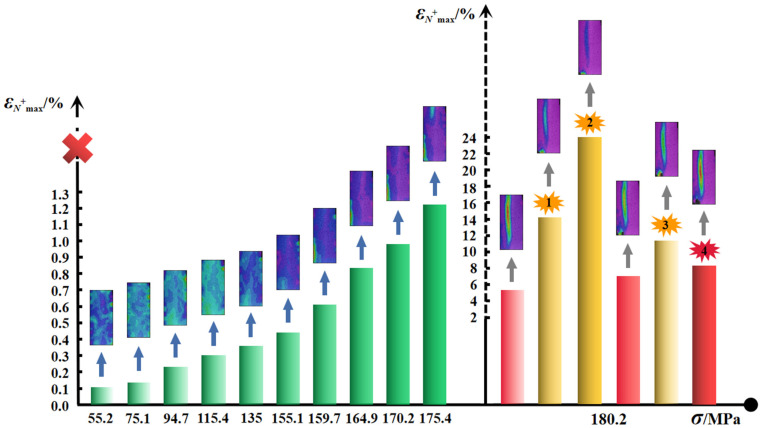
Positive maximum of net strain with stress level for granite.

**Figure 9:**
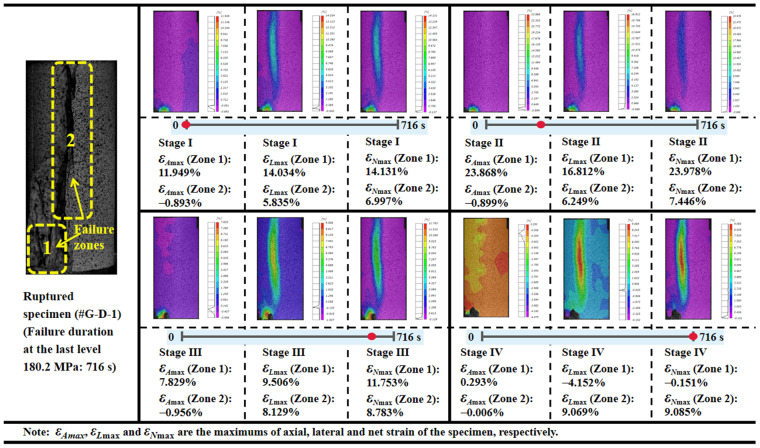
Maximum deformation of granite in failure areas during the last stress stage (180.2 MPa).

From the view of the net strain that can also be deemed as another description form of volumetric deformation, the evolution of the positive maximum, which refers to the greatest magnitude of dilation with the associated stress level, is presented in Figure 8. The volumetric dilation gradually increases until it governs the deformation to the ultimate rupture at 180.2 MPa. Four clear fracture sounds are recorded, and two main failure regions emerge during the last stress stage, which lasts for 716 s (see Figure 8 and Figure 9). As the applied load begins with 94.7 MPa, the axial deformation in the bottom-left corner of the #G-D-1 constantly increases until the first rupture occurs (Failure Zone 1), the distinct similar change for the lateral strain commences with 159.7 MPa, and *ε_A_* and *ε_L_* increase by −0.385% and 0.632%, respectively, compared to the case at 175.4 MPa. In the meantime, a sign of creep failure has been also shown by the lateral extension during the loading stage for 175.4 MPa and the maximum of *ε_L_*_main_ is 0.482%. The biggest ruptured area (Failure Zone 2) then forms, close to the middle of the specimen, and the main fracture plane fully penetrates the rock accompanying a loud and sharp crack. It can be found that, before Stage Ⅳ, the deformation is more marked in Failure Zone 1 and the difference of the maximum volumetric dilation magnitude is more than triple. Thus, despite being difficult to precisely tell the sound and the corresponding ruptured location, it can be deduced that the first few cracking sounds may well derive from the first failure region.

### 3.5. Characterization of Time-Dependent Properties by AE Technology

#### 3.5.1. Complete Specimen

**Figure 10:**
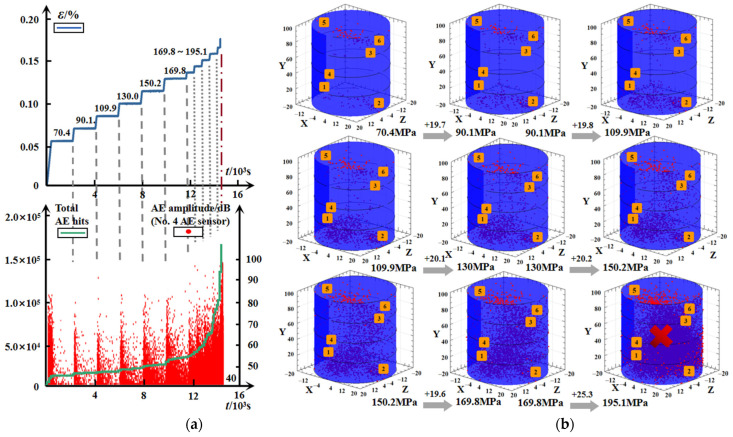
Time-dependent evolution of AE activities for granite. (**a**) AE hits and AE amplitude. (**b**) Spatial development of AE event points.

For the specimen #G-D-2, Figure 10 displays the time-dependent comparison between the deformation and the real-time AE characteristic parameters, including the number of AE hits calculated from all six sensors, and the representative AE amplitude extracted from one of the AE recording channels (No. 4), as well as the spatial evolvement of the AE event points with the applied load. The recorded curve of the accumulated AE hits exhibits the similar behavior to the strain during the whole creep process, and the three phases regarding the instantaneous elastic deformation, attenuation, and stabilization are more evident. At the initial loading before reaching each constant stress level, the notable AE signals mainly originate from the closure and friction of the original micro-cracks. Then AE activities decline rapidly when the applied load remains unchanged. The attenuation pattern continues during the low stress stages, and it gradually enhances with the stress level, which can be explained by the dislocation theory, that due to the heterogeneity of rock, the residual stress is generated by the non-uniform displacements at both ends of a slip face [27], resulting in the self-adjustment inside the structure that causes the propagation of the original crack or the initiation of new crack. The residual stress decreases by the action of micro-crack growth, and the rock maintains a relatively balanced state corresponding to the stable stage of the AE amplitude evolution. With the further increase in the applied stress, more micro-crack generations and propagations are triggered by the local heterogeneous failure, owing to the partial internal stress surpassing the yield strength of rock material. The attenuation trend fades, and irregular variations in the AE activities appear. The AE amplitude is even higher after the decay, marking the time at which the specimen enters the unstable stage and failure is approaching. As the rock is broken, both the AE hits and the AE amplitude attain the maximum within an extremely short period.

The spatial evolution of the AE event points during the multistage loading is presented in Figure 10b, where numbers 1–6 represent the center position for the contact surface of the AE sensor, and the red dot represents the AE event point recorded in a 3D system of coordinates. The evolution, comprising the change of density and the distribution of the AE event points, reflects the initiation and development of micro-cracks in the interior of the specimen. Compared to Figure 10a, the AE event points are initially almost close to both ends of the specimen, and they slightly increase between the first and second creep loading (70 to 90 MPa) (Figure 10b), suggesting that small amounts of damage accumulate at a low stress level, which is attributed to the compaction on the micro-cracks and the specimen returning to a steady state through the adjustment and redistribution of internal stress. With the applied increase in stress, the inner stability of the rock no longer stays, and this unbalance leads to the prominent rise in the micro-crack density by the accelerated propagation and intensive coalescence, until the accelerating creep stage, where the macro-fissures come up accompanied by a number of AE event points, marking that the internal damage accumulation has exceeded the threshold to break the structure integrity of the specimen.

**Figure 11:**
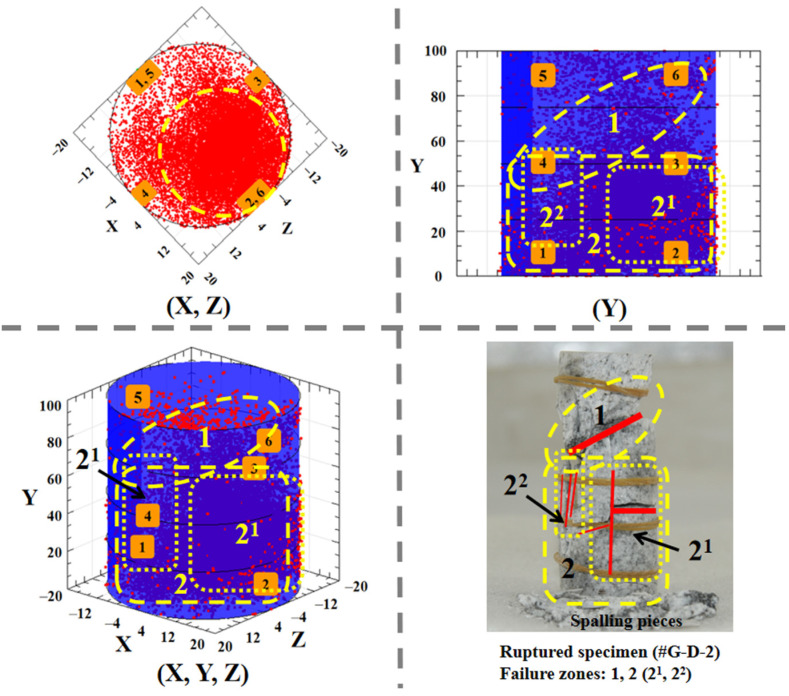
Failure areas and corresponding damage accumulation inside intact specimen of granite.

Corresponding to the spatial distribution of the AE event points from multiple angles, the specimen #G-D-2 that is in the form of the fracture is presented in Figure 11. The specimen is divided into the two main ruptured parts and the perspective on the 1D coordinate (Y) and the 3D coordinates (X, Y, Z) is more visible. It is observed that the top region is more damaged, resulting from the largest and penetrated shear plane, and the bottom fractured area is bigger with various scattered local splitting failures. The results indicate that the space positioning of the AE activity is well in line with the failure zone of the rock.

#### 3.5.2. Incomplete Specimen

**Figure 12:**
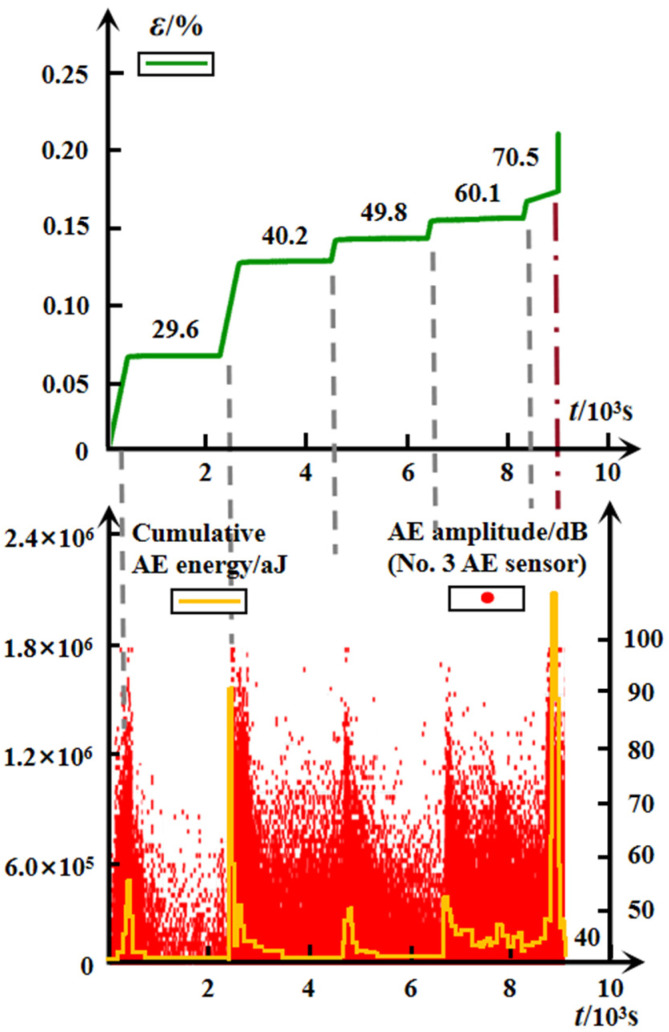
Time-dependent evolution of AE energy and AE amplitude for defective specimen of granite.

**Figure 13:**
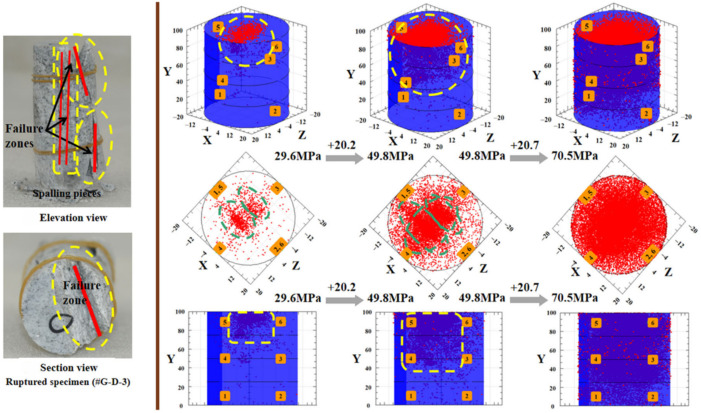
Failure areas and corresponding damage accumulation inside defective specimen of granite.

As a defective specimen, some surface fissures can be observed with the naked eye. The time-dependent variation of the cumulative AE energy of the six attached AE sensors and the typical AE amplitude, recorded by the No. 3 AE channel, and the multistage spatial evolution of the AE event points of #G-D-3, are shown in Figure 12 and Figure 13, respectively. Both the AE cumulative energy and the AE amplitude exhibited the similar attenuation trend to the #G-D-2 before the unsteady phase at the last two applied stresses. Except for the last stress stage, the AE activity is in the most active state during the initial loading of the second level (40.2 MPa), which can be predicted by the earlier several brisk noises and the significant distribution of the AE event points on the top region of the specimen at 29.6 MPa (see Figure 13). It can be speculated that the local failure area may have been formed inside the rock because of the swift aggregation of internal damage that is heavily induced by the growth of the original micro-defects. For the incomplete specimen, the random distribution of the initial micro-defects brings about the inhomogeneity of rock to a great degree, which is another cause of the local internal imbalance and failure. Besides, the section view of the ruptured specimen #G-D-3 suggests that the fracture may likely occur in the top area in the first place, based on the process from the AE event points intensively aggregating at a low stress level until it eventually becomes full of the rock.

According to the above analysis, due to the striking damage accumulation inside the specimen produced by the faster and more dramatic evolution of micro-cracks, which is reflected by the denser spatial distribution of the AE event points in real-time, the failure strength of the #G-D-3 is much lower than the #G-D-2, and the deformation is more conspicuous in the former.

### 3.6. Creep Failure Mode

**Figure 14:**
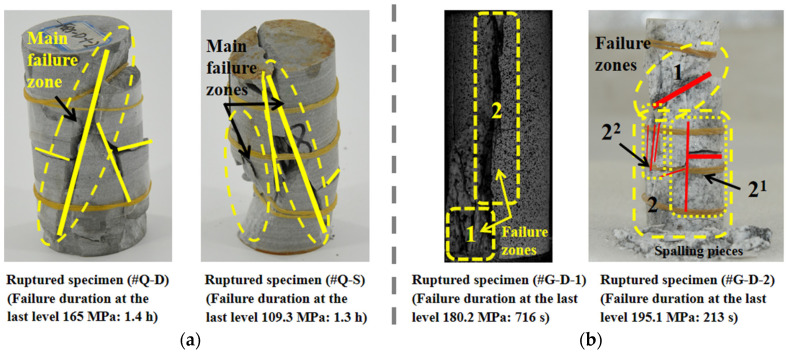
Failure specimens of quartz sandstone and granite. (**a**) Quartz sandstone. (**b**) Granite.

The fractured rock specimens of quartz sandstone and granite are exhibited in Figure 14a,b, respectively. The dry specimen #Q-D shows that the main shear zone is complete via both ends of the rock, accompanied by the local splitting failure with a few less palpable tensional cracks and spalling pieces. The combination of the shear and splitting failures are shown by the saturated specimen #Q-S, whose shear region is bigger. The inner damage accumulation causes the increase in the anisotropy of rock and the local failure region that appears after reaching the yield strength, producing the more cumulative damage, which results in the lasting extension of the failure range under the time effect. Furthermore, under the interaction between moisture and hydrophilic matter, the stability of structure declines in the interior of the rock, and with the increasing load level, the tensional stress along the axial direction arises after the stress redistribution, which finally gives rise to the macroscopic tensional cracks.

For the granite, although the difference in the failure pattern is relatively evident, where #G-D-1 and #G-D-2 separately present the main tensile splitting type and the coexisting shear and splitting failure, which may be closely related to the heterogeneity of the structure and the composition of the rock, both specimens fail in a rheological manner with the ductile damage accumulation.

## 4. Conclusions

From the various perspectives, the time-dependent characteristics of deep brittle rocks, including quartz sandstone and granite, are investigated by the multilevel-uniaxial creep experiments combined with the DIC and AE technologies in real time, and the conclusions in this study are drawn as follows:

1. During the evolvement process from the time-dependent deformation to the failure of quartz sandstone, the lateral strain is more sensitive to the increasing applied load and the lateral ductility is more significant.
2. The quartz sandstone, in a saturated state, shows a certain bearing capacity before the volumetric dilation predominance, and under the softening effect caused by the moisture, the Poisson’s ratio maintains a nearly constant value during the middle stress stages. The more notable increasing tendency of the steady creep rate with the increasing stress level is exhibited by the larger slope and intercept in the fitting relations.
3. Compression and extension coexist on the granite during the creep process. The failure may first occur in the prominent deformation area and may correspond to the initial cracking sound.
4. The AE hits present the similar time-dependent behaviors to the deformation of the granite, and both the AE amplitude and energy display the attenuation mode before the rock enters into the unstable stage. The spatial distribution of the AE event points reflect that the defective rock produces the more remarkable damage accumulation, which induces lower strength and larger deformation than the intact rock.
5. The dry and saturated quartz sandstone, respectively, show the main shear pattern and the coexistence of the shear and splitting failures. However, since the failure mode of the granite is relatively manifold, detailed research on its formation mechanism will be conducted in future work.

## Figures and Tables

**Table 1:** *C*_3_/*r*_c_^1/2^ and In*a*(H_2_O) + *C*_4_ in axial and lateral fitting relationships of dry and saturated quartz sandstone specimens.

	#Q-S (Axial)	#Q-S (Lateral)	#Q-D (Axial)	#Q-D (Lateral)
*C*_3_/*r*_c_^1/2^	0.034	0.0459	0.0248	0.0326
ln*a*(H_2_O) + *C*_4_	−22.7965	−23.5382	−23.4926	−23.594
*R* ^2^	0.8781	0.9412	0.8502	0.962

## Data Availability

The data presented in this study are available on request from the corresponding author.
